# Heat shock proteins in long-lived worms and mice with insulin/insulin-Like signaling mutations

**DOI:** 10.18632/aging.100058

**Published:** 2009-06-15

**Authors:** William R. Swindell

**Affiliations:** University of Michigan, Departments of Pathology and Geriatrics, Ann Arbor MI 48109, USA

**Keywords:** Aging, chaperone, growth hormone, longevity, Snell, stress

## Abstract

Heat shock proteins (HSPs) have proven to be effective tools for extending invertebrate lifespan, and inC. elegans daf-2 mutants, longevity resulting from loss of insulin / insulin-like signals is at least partly dependent upon elevated HSP expression. In mice, inhibition of the orthologous growth hormone / insulin-like growth factor I (GH / IGF-I) pathway has similar pro-longevity effects. A recent study, however, suggests that loss of GH / IGF-I signals in long-lived mice does not broadly elevate HSP expression, but in fact decreases HSP expression in many tissue types, such as liver and kidney. The contribution of chaperones to the longevity of long-lived mice with altered GH / IGF-I signals may therefore differ from that described in C. elegans daf-2 mutants. This result, in combination with other recent findings, underscores the possibility that systemic overexpression of chaperones will have dissimilar effects on longevity in vertebrate and invertebrate systems.

Heat shock proteins (HSPs) have gained prominence in aging research and are thought to be of importance for both longevity and overall maintenance of proteome integrity with advancing age. The most compelling evidence so far has been collected from invertebrate models, in which it was found that longevity and thermotolerance were joint effects of certain mutations [1,2], and that brief exposure to mild heat stress at a young age increased both HSP expression and organismic lifespan [3]. These investigations prompted studies of specific HSPs and their effects, and it was soon discovered, in *D. melanogaster*, that the hormesis effect of mild heat shock on survival was enhanced within transgenic strains carrying extra copies of the *Hsp70* gene [4]. These results served to spark a broader interest in HSPs and their possible role in aging pathology, and it was soon found that systemic and lifelong overexpression of HSP-encoding genes could increase lifespan in *Drosophila* [5-8], as well as *C. elegans* [9-11]. The relevance of these observations to basic aging research was reinforced by the finding that loss of insulin / insulin-like signaling (IIS), the most robust pro-longevity genetic manipulation currently known, partly influenced longevity by modulating HSP expression, particularly the low molecular weight small HSPs [10,12,13]. Most notably, Hsu et al. [12] showed that expression of *hsp-16.1*, *hsp-16.49*, *hsp-12.6* and *sip-1* were all elevated in *daf-2* mutant worms, and that expression of each HSP was required for the full effect of *daf-2* on lifespan. Taken together, these observations, appeared consistent with a "chaperone overload" theory of aging [14], which proposed that HSP activity was critical for maintenance of proteostasis, such that increased HSP abundance could indeed contribute to lengthened lifespan and reduction of age-related disease [15].

While efforts to increase invertebrate lifespan by augmenting HSP abundance have often been successful, it has not been demonstrated that systemic and lifelong HSP overexpression can extend mammalian lifespan. However, the overexpression of certain HSPs can ameliorate specific types of age-related pathology in laboratory rodents, including decline in muscle function [16], accumulation of toxic tau aggregates in neural tissue [17], development of insulin resistance [18], oxidative stress damage [19], and activation of inflammatory pathways [20], suggesting that results from invertebrate studies can indeed provide a useful guide for exploring the involvement of HSPs in vertebrate aging, and possibly, longevity. In bridging the gap between invertebrate and vertebrate models, the regulation of HSP expression by IIS provides a good starting point, given that the IIS pathway has effects on lifespan and a broad spectrum of age-related diseases that are in fact conserved across species [21]. In the *C. elegans* model, loss of IIS increases expression of small HSPs [12,22], as well as HSPs of larger size [23,24], possibly by enhancing binding of both daf-16 (FOXO) and HSF to promoter regions of HSP-encoding genes [12]. Each of these molecular components has orthologous elements in mice, although the mammalian system also differs in some key respects. In particular, circulating IGF-I levels in mice are linked to GH, such that both hormones are considered part of the same GH / IGF-I endocrine axis. Despite this difference, however, loss of GH / IGF-I signaling in mice has effects on lifespan that parallel loss of IIS in *C. elegans* and *Drosophila*, as exemplified by a series of long-lived dwarf mouse models, including the GH-deficient *Pit1*(*dw*/*dw*) ("Snell") and *Prop1*(*df*/*df*) ("Ames") mice, the GH-insensitive *Ghr*(-/-) mice, and the *PappA*(-/-) mouse with localized reductions in IGF-I signaling [25]. It is therefore intriguing to ask whether such mutant mice, like the *C. elegansdaf-2* mutants, have elevated HSP expression levels, and whether such HSP expression patterns contribute to their aging-related phenotypes, as well as to their stress resistance properties [26].

## Regulation of HSP expression by endocrine signals in long-lived mice

The chaperone activity of HSPs is critical for recovery from stress and cellular insults, but HSPs have diverse functions that are of importance even in the absence of an external stress condition, including cellular transport, peptide synthesis and modulation of key cell signaling pathways [20,27]. It is hardly surprising, in this sense, that systemic mechanisms would have evolved to enable precise control of the abundance and distribution of HSPs across major organ systems, and that this control might be accomplished through a circulating neuroendocrine factor. With these expectations in mind, and in view of the known effects of IIS on HSP expression in *C. elegans*, Swindell et al. [28] have recently examined the effects of the *Pit1*(*dw*/*dw*) and *Ghr*(-/-) mutations on HSP expression patterns in six different tissue types in the laboratory mouse (liver, kidney, heart, lung, muscle and neocortex). These mutations were of interest, since both lead to reduced IGF-I levels in circulation, as well as increased lifespan, presumably through mechanisms that would parallel those associated with the *C. elegans daf-2* mutation. Surprisingly, however, effects of these mutations on gene expression varied among HSP-encoding genes, among tissue types, and with little unifying pattern. Rather than having widely elevated HSP expression levels, long-lived dwarf mice were instead a mosaic of both elevated and depressed HSP mRNAs, depending upon the tissue examined and the particular HSP target evaluated [28]. The specific role of GH / IGF-I in these expression patterns was evaluated by comparisons between *Pit1*(*dw*/*dw*) and *Ghr*(-/-) mutants, and in general, it was only the declines of HSP expression resulting from *Pit1*(*dw*/*dw*) that were also shared by *Ghr*(-/-), particularly in liver and kidney. Unexpectedly, therefore, these results suggested that expression of some HSPs was positively related to GH / IGF-I signals, and this notion was further supported by the observation that a six-week treatment of GH injections increased hepatic expression of *Hsph1* (*Hsp110*), *Hspa5* (*Bip*),*Dnajb11* and *Dnajc3* (*p58IPK*) in the GH-deficient Ames dwarf mouse [28]. The picture that has emerged from these studies, therefore, is one of complex HSP regulation in vivo, with GH / IGF-I either indirectly or directly acting to modulate the expression of at least some HSP-encoding genes, but no generalized or systemic induction of HSP expression in long-lived dwarf mice.

Should we conclude that the *C. elegans* system provides a poor mammalian analogue for investigating the contributions of HSPs to age-related pathology, and that alterations in HSP expression contribute to increased survivorship of *daf-2* mutants in a fashion that is unparalleled in long-lived dwarf mice? Certainly not. Above all else, it is clear that, in mice, broad and general conclusions regarding the interaction between HSPs and longevity mutations are apt to be hazardous. This reflects, in part, the fact that there is considerable diversity among HSPs, both in terms of function and their regulation, such that only subsets of certain HSPs are likely to be co-regulated in vivo. On balance, there appears to be a stronger tendency for loss of GH / IGF-I signaling to decrease HSP expression in mice, rather than to increase it, but some patterns are in line with the *C. elegans* model. For instance, the alpha-crystallin HSP *Cryab*, which is orthologous to the *C. elegans* Hsp-16 small HSPs [29], is increased by *Pit1*(*dw*/*dw*) in cardiac tissue, and two other small HSP genes (*Hspb7* and *Hspb8*) were increased by both *Pit1*(*dw*/*dw*) and *Ghr*(-/-) in lung [28]. Another important consideration is that, in *daf-2* mutants, differences in HSP expression between mutant and wild-type worms are amplified by an external stress condition [12,22], suggesting the involvement of stress-activated regulatory factors, such as heat shock factor (HSF). It is indeed possible that, in long-lived dwarf mice, although basal HSP expression is generally lower, this is perhaps associated with a more robust heat shock response, such that under a stress condition, loss of GH / IGF-I signals would serve to augment HSP expression [30]. While intriguing, this idea has not, so far, been supported by investigations of HSP stress-response patterns in fibroblast cells from long-lived *Pit1*(*dw*/*dw*) mice [28,31].

We can be assured that further work will reveal more impressive similarities, and differences, between the influence of mouse and *C. elegans* IIS longevity mutations on HSPs, both in terms of HSP function and transcriptional regulation. Interestingly, for example, it was recently found that mutant *C. elegans* worms lacking the AFD sensory neuron exhibited an altered heat shock response in somatic cells, even though such cells were not directly innervated by this sensory neuron [32]. In worms, therefore, machinery regulating HSP expression is partly under neuronal control, and it appears that a type of neuroendocrine signal mediates between sensory neurons and somatic cells to allow control of HSP expression under stress [32]. It will be of much interest to determine whether there exists parallelism between such, as yet unidentified, neuroendocrine signals in *C. elegans*, and the regulation of HSP expression by GH / IGF-I signals in mice. At the same time, it will be illuminating to develop and clarify a model by which HSP expression is regulated by GH / IGF-I signals in mice. For instance, it is necessary to understand whether GH / IGF-I signals are a direct modulator of HSP expression, and if not, which intermediate pathways act as secondary signals. It is indeed possible that effects of GH / IGF-I signals on in vivo HSP expression are very indirect, and mediated by metabolic responses to circulating GH / IGF-I (e.g. increased muscle mass, improved insulin sensitivity). It will also be important to evaluate whether effects of GH / IGF-I on HSP expression are dependent upon HSF-1, although at present, the complex shifts in HSP expression documented in GH / IGF-I mutants suggest that a single transcription factor will not account for all modulation of HSP transcription. Lastly, given that GH / IGF-I signals do modify HSP expression in mice, the possibility of complex feedback loops should be explored, by which HSP abundance might influence GH or IGF-I signals. Along these lines, transgenic mice overexpressing Hsp70 exhibit a growth deficit and a 50% reduction in serum IGF-I levels [33], Hsp60 augments IGF-I signals by inhibiting degradation of the IGF-I receptor in cardiac tissue [34], and in cartilage, Hsp90 appears to facilitate IGF-I-induced phosphorylation of AKT [35].

## What's next? Can we "chaperone our way" to extended lifespan in mice?

Establishing the contribution of HSPs to longevity in GH / IGF-I mutant mice, and whether this contribution parallels that described in *daf-2* worms, will shed light on the more general issue of how HSPs might influence lifespan and age-related disease in mammalian systems. Clearly, HSP overexpression has, in many but not all cases, been successful as a pro-longevity manipulation in invertebrate models, presumably due to enhanced proteostasis with age and/or protection against oxidative stress damage [15]. It would, at present, be valuable to obtain a comparable success story involving laboratory rodents, which would establish that chaperones can be used as a pro-longevity therapeutic in mammals. Systemic and lifelong HSP overexpression (or elevated HSF-1 activity) seems unlikely to be a winning strategy, since there is now considerable evidence that this can increase mortality in organisms for which cancer figures prominently in the disease spectrum [33, 36 - 38]. Moreover, if we are to learn from the two manipulations most reliably known to increase mouse lifespan, loss of GH / IGF-I signals and dietary restriction, we'd walk away the notion that, at a systemic level, decreased expression of certain HSP-encoding genes (e.g., *Hsph1*) is associated with longevity more so than increased HSP expression [28,39]. In consideration of this, it appears that modification of existing thought paradigms will be required for exploring the role of HSPs as longevity therapeutics, perhaps in combination with more nuanced genetic or pharmacological approaches. In some mitotic tissues, inhibition of HSF-1 activity or decreased HSP abundance may serve to combat malignant transformation [37]. On the other hand, this may be deleterious in tissues with post-mitotic cell populations, such as heart and the central nervous system [40,41], where results generated from invertebrate models are more likely to be applicable in direct fashion, such that manipulations augmenting HSP activity might positively affect lifespan or certain disease outcomes. A good exemplar of this approach is the study of Li and Ren [42], in which cardiac-specific overexpression of IGF-I led to a small but significant increase in mouse lifespan, even though the anti-apoptotic effects of this manipulation would likely have limited organismic survival if it were applied systemically or to other organ systems. Similar tissue-specific strategies may be appropriate for evaluating whether enhanced HSP abundance is a viable pro-longevity strategy in mice, and indeed, cardiac tissue may provide a suitable domain for future investigations utilizing this line of attack [41, 43-46].
